# Fenômeno de Fluxo Lento Coronariano - Adicionando Fibrose Miocárdica à Equação

**DOI:** 10.36660/abc.20200187

**Published:** 2020-04-06

**Authors:** Filipe Penna de Carvalho, Clério Francisco de Azevedo

**Affiliations:** 1 Diagnósticos da América SARio de JaneiroRJBrasilDiagnósticos da América SA, Rio de Janeiro, RJ - Brasil; 2 Américas Serviços MédicosRio de JaneiroRJBrasilAméricas Serviços Médicos, Rio de Janeiro, RJ - Brasil; 3 Duke University HospitalDurhamNorth CarolinaEUADuke University Hospital - Medicine/Cardiology, Durham, North Carolina – EUA

**Keywords:** Insuficiência Cardíaca, Reserva Fracionada de Fluxo Miocárdio, Cicatriz Hipertrófica, Prognóstico, Peptídeo Natriurético Tipo C, Fibrose Endomiocárdica, Espectroscopia de Ressonância Magnética/métodos

Inicialmente descrito há mais de 40 anos por Tambe et al.,^1^o fenômeno de fluxo lento coronariano (FLC) é caracterizado por retardo da progressão do meio de contraste na ausência de doença epicárdica coronariana obstrutiva durante angiografia coronária invasiva (ACI).^2^ O FLC afeta tipicamente jovens fumantes do sexo masculino, que frequentemente apresentam síndrome coronariana aguda (SCA) ou angina de repouso refratária recorrente que requer internação.^2-4^ Além disso, arritmias potencialmente fatais e morte súbita cardíaca também foram associadas ao FLC.^5^

Apesar do aumento da conscientização e da pesquisa, o FLC permanece uma condição difícil de descrever e pouco compreendida, com muitos mecanismos patogênicos propostos, incluindo disfunção endotelial, vasomotora e microvascular.^2,6^ De fato, uma regulação anormal do tônus microvascular que ocorre apenas durante condições de repouso, enquanto a reserva de fluxo coronariano está dentro da faixa normal, foi descrita no FLC.^7^

Nessa edição dos Arquivos Brasileiros de Cardiologia, Candemir et al.,^8^ dão uma contribuição importante a esse campo do conhecimento. Os autores estudaram 35 pacientes com dor torácica encaminhados para uma angiografia coronária invasiva (ACI) diagnóstica. Todos tinham níveis negativos de troponina e nenhuma evidência de isquemia no teste ergométrico. Foi feita uma comparação entre os pacientes que apresentaram FLC na artéria descendente anterior esquerda (n = 19) e os controles pareados com artérias coronárias normais e sem anormalidades do fluxo coronariano (n = 16). Eles procuraram investigar se as cicatrizes no miocárdio identificadas por ressonância magnética cardíaca (RMC) e / ou se os níveis do fragmento N-terminal do peptídeo natriurético tipo B (NT-ProBNP) eram mais frequentes no grupo com FLC. É importante ressaltar que, pelo que sabemos, este foi o primeiro estudo a utilizar a RMC para avaliar a presença de fibrose miocárdica na população com FLC.

Vale ressaltar que a RMC utilizando a técnica de realce tardio é agora uma ferramenta amplamente disponível e poderosa que permite a identificação e quantificação precisa da fibrose miocárdica, com vários estudos demonstrando sua utilidade no diagnóstico e prognóstico de cardiomiopatias tanto isquêmicas quanto não isquêmicas.^9-15^ Curiosamente, os autores demonstram que o realce tardio estava presente em até 52,5% (n = 10) dos pacientes com FLC, em oposição a nenhum no grupo controle. Os autores concluíram que o FLC pode resultar em alterações irreversíveis no tecido miocárdico.

Entretanto, não acreditamos que os dados apresentados possam ser utilizados para estabelecer causalidade entre FLC e fibrose miocárdica. Por exemplo, em um subgrupo de pacientes (n = 3), a lesão miocárdica foi observada nas paredes inferior e ínfero-lateral, e não no território típico da artéria coronária descendente anterior esquerda, onde estava presente o FLC. Além disso, os autores não descrevem se o padrão de realce tardio observado em seu estudo era predominantemente isquêmico (por exemplo, subendocárdico ou transmural) ou não-isquêmico (mesocárdico ou epicárdico). Mais importante ainda, como os autores apontam durante a discussão, eles realizaram um estudo transversal e, portanto, nenhuma relação temporal pode ser estabelecida entre o FLC e a fibrose miocárdica. Uma das muitas explicações possíveis é que esses pacientes com fibrose miocárdica na RMC podem ter apresentado anteriormente um infarto do miocárdio com artérias coronárias normais (MINOCA, do inglês *myocardial infarction with normal coronary arteries*) e o FLC é apenas uma consequência desse evento anterior. Embora os autores tenham encontrado uma associação entre o FLC e a fibrose miocárdica, acreditamos que são necessários mais estudos para determinar se existe uma relação causal entre eles.

Curiosamente, níveis mais altos de NT-pro-BNP, um conhecido marcador prognóstico na SCA,^16^ também foram observados em pacientes com FLC quando as cicatrizes miocárdicas foram detectadas por RMC, em comparação com o FLC sem evidência de fibrose na RMC (NT-pro- BNP = 147,10 pg / mL vs. 28,0 pg / mL, p = 0,03).

É importante ressaltar que, em um estudo publicado anteriormente por Yurtdaş et al.,^6^ demonstrou-se que níveis elevados de NT-pro-BNP se correlacionaram com angina e infradesnivelamento do segmento ST em pacientes com FLC durante testes em esteira. No entanto, nenhuma anormalidade foi observada durante o teste ergométrico em nenhum paciente deste estudo. Novamente, embora os autores demonstrem uma associação de NT-pro-BNP com FLC e fibrose miocárdica, acreditamos que nenhuma relação causal definitiva possa ser estabelecida com base nos dados apresentados.

Em geral, este é um trabalho interessante de Candemir et al.,^8^ utilizando a RMC para estudar uma condição ainda obscura. Achamos muito interessante que a RMC tenha permitido a detecção de fibrose miocárdica em um subgrupo de pacientes com FLC sem história prévia de infarto do miocárdio. A identificação de cicatrizes miocárdicas utilizando imagens de realce tardio é uma poderosa ferramenta prognóstica em múltiplas cardiomiopatias, isquêmicas e não isquêmicas. Embora pequeno em tamanho, este estudo de Candemir et al.,^8^ abre novas possibilidades de pesquisa para responder se há causalidade na associação entre FLC e fibrose miocárdica e se a presença de fibrose miocárdica nesses pacientes tem alguma implicação prognóstica ou se, por exemplo, está associada a uma maior probabilidade de arritmias malignas. Por outro lado, pesquisas futuras utilizando novas técnicas de RMC para caracterização de tecidos, incluindo o mapeamento de T1 e T2, também podem ajudar a esclarecer essa condição ainda pouco compreendida.
