# Supplementary material for: Fenômeno de Fluxo Lento Coronariano - Adicionando Fibrose Miocárdica à Equação
Source: Arq Bras Cardiol. 2020 Apr 6;114(3):552–3. [Article in Portuguese] doi: 10.36660/abc.20200187 (PMC7792736; doi:10.36660/abc.20200187)
Supplement: Translation

# Translation: Coronary Slow Flow Phenomenon - Adding Myocardial Fibrosis to the Equation

**Authors:** Filipe Penna de Carvalho, Clério Francisco de Azevedo

**Affiliations:** 1 BrazilDiagnósticos da América SA, Rio de Janeiro, RJ - Brazil; 2 BrazilAméricas Serviços Médicos, Rio de Janeiro, RJ - Brazil; 3 USADuke University Hospital - Medicine/Cardiology, Durham, North Carolina – USA

**Keywords:** Heart Failure, Fractional Flow Reserve, Cicatrix, Hypertrophic, Prognosis, Natriuretic-Peptide C-Type, Endomyocardial Fibrosis, Magnetic Resonance Spectroscopy

Initially described more than 40 years ago by Tambe et al.,^1^ coronary slow flow phenomenon (CSFP) is characterized by delayed contrast medium progression in the absence of obstructive coronary epicardial disease during invasive coronary angiography.^2^ CSFP typically affects young male smokers, who often presents with acute coronary syndrome (ACS) or recurrent refractory resting angina requiring hospital admission.^2-4^ Moreover, life-threatening arrhythmias and sudden cardiac death have also been associated with CSFP.^5^

Despite increased awareness and research, CSFP remains an elusive and poorly understood condition, with many proposed pathogenic mechanisms including endothelial, vasomotor and microvascular dysfunction.^2,6^ Indeed, an abnormal regulation of microvascular tone that occurs only during resting conditions, while coronary flow reserve is within normal range, has been described in CSFP.^7^

In this issue of *Arquivos Brasileiros de Cardiologia*, Candemir et al.,^8^ add an important contribution to this field of knowledge. The authors studied 35 patients with chest pain referred for a diagnostic invasive coronary angiography (ICA). All had negative troponin levels and no evidence of ischemia on exercise stress testing. A comparison was made between patients who presented with CSFP in the left anterior descendant artery (n=19) and matched controls with normal coronary arteries and no coronary flow abnormalities (n = 16). They sought to investigate if myocardial scarring identified using cardiac magnetic resonance imaging (CMR) and/or if N-terminal Pro B-type Natriuretic Peptide (NT-Pro-BNP) levels elevation were more frequent in the CSFP group. Importantly, to the best of our knowledge, this was the first study to use CMR to evaluate the presence of myocardial fibrosis in the CSFP population.

Noteworthy, CMR using the delayed enhancement (or late gadolinium enhancement) technique is now a widely available and a powerful tool that allows the precise identification and quantification of myocardial fibrosis, with multiple studies demonstrating its utility in the diagnosis and prognosis of both ischemic and non-ischemic cardiomyopathies.^9-15^ Interestingly, the authors demonstrate that delayed enhancement was present in up to 52.5% (n = 10) of the patients with CSFP as opposed to none in the control group. The authors thus concluded that CSFP may result in irreversible changes in the myocardial tissue.

However, we do not believe the presented data can be used to establish causality between CSFP and myocardial fibrosis. For instance, in a subset of patients (n = 3) the myocardial injury was seen in the inferior and inferolateral walls, and not in the typical left anterior descendant coronary artery territory where CSFP was present. Moreover, the authors do not describe whether the delayed enhancement pattern observed in their study was predominantly ischemic (e.g., subendocardial or transmural) or non-ischemic (midwall or epicardial). Most importantly, as the authors point out during the discussion, they performed a transversal study and, thus, no temporal relationship between CSFP and myocardial fibrosis can be established. One of the many possible explanations is that these patients with myocardial fibrosis by CMR might have previously presented with a myocardial infarction with normal coronary arteries (MINOCA) and the CSFP is but a consequence of this previous event. Although the authors did find an association between CSFP and myocardial fibrosis, we believe that further research is needed to determine whether there is a causal relationship between them.

Interestingly, higher NT-pro-BNP levels, a well-known marker of prognosis in ACS,^16^ were also seen in patients with CSFP when myocardial scarring was detected by CMR, as compared to CSFP without evidence of fibrosis by CMR (NT-pro-BNP = 147.10 pg/ml vs . 28.0 pg/ml, p = 0,03).

Importantly, in a previously published study by Yurtdaş et al.,^6^ elevated NT-pro-BNP levels were shown to correlate with angina and ST-segment depression in patients with CSFP during exercise treadmill testing. However, no abnormalities were seen during exercise testing in any patient of this study. Again, although the authors demonstrate an association of NT-pro-BNP with CSFP and myocardial fibrosis, we believe that no definitive causal relationship can be established based on the presented data.

Altogether, this an interesting work by Candemir et al.^8^using CMR to study a still obscure condition. We find it very interesting that CMR allowed the detection of myocardial fibrosis in a subgroup of patients with CSFP without history of prior myocardial infarction. Myocardial scarring identification using delayed enhancement imaging is a powerful prognostic tool in multiple cardiomyopathies, both ischemic and nonischemic. Although small in size, this study by Candemir et al.^8^ opens up new research possibilities to answer whether there is causality in the association between CSFP and myocardial fibrosis and if the presence of myocardial fibrosis in these patients have any prognostic implication or, for instance, is associated with a higher likelihood of malignant arrhythmias. Conversely, future research using novel CMR techniques for tissue characterization, including T1 and T2 mapping, may also help shed light into this still poorly understood condition.
